# Oral health in relation to all-cause mortality: the IPC cohort study

**DOI:** 10.1038/srep44604

**Published:** 2017-03-15

**Authors:** Margaux Adolph, Christelle Darnaud, Frédérique Thomas, Bruno Pannier, Nicolas Danchin, G. David Batty, Philippe Bouchard

**Affiliations:** 1 Department of Periodontology, Service of Odontology, Rothschild Hospital, AP-HP, Paris 7-Denis Diderot University, U.F.R. of Odontology, Paris, France; 2 Centre d’Investigation Préventive et Clinique (IPC), Paris, France; 3 Manhès Hospital, Fleury-Mérogis, France; 4 Department of Cardiology, Georges Pompidou European Hospital, AP-HP, Paris 5 - Descartes University, Medicine Faculty, Paris, France; 5 Department of Epidemiology and Public Health, University College London, London, UK; 6 EA 2496, Paris 5 - Descartes University, U.F.R. of Odontology, Paris, France

## Abstract

We evaluated the association between oral health and mortality. The study population comprised 76,188 subjects aged 16–89 years at recruitment. The mean follow-up time was 3.4 ± 2.4 years. Subjects with a personal medical history of cancer or cardiovascular disease and death by casualty were excluded from the analysis. A full-mouth clinical examination was performed in order to assess dental plaque, dental calculus and gingival inflammation. The number of teeth and functional masticatory units <5 were recorded. Causes of death were ascertained from death certificates. Mortality risk was evaluated using Cox regression model with propensity score calibrated for each oral exposure. All-cause mortality risk were raised with dental plaque, gingival inflammation, >10 missing teeth and functional masticatory units <5. All-cancer mortality was positively associated with dental plaque and gingival inflammation. Non-cardiovascular and non-cancer mortality were also positively associated with high dental plaque (HR = 3.30, [95% CI: 1.76–6.17]), high gingival inflammation (HR = 2.86, [95% CI: 1.71–4.79]), >10 missing teeth (HR = 2.31, [95% CI: 1.40–3.82]) and functional masticatory units <5 (HR = 2.40 [95% CI 1.55–3.73]). Moreover, when ≥3 oral diseases were cumulated in the model, the risk increased for all-cause mortality (HR = 3.39, [95% CI: 2.51–5.42]), all-cancer mortality (HR = 3.59, [95% CI: 1.23–10.05]) and non-cardiovascular and non-cancer mortality (HR = 4.71, [95% CI: 1.74–12.7]). The present study indicates a postive linear association between oral health and mortality.

Oral health status is denoted by a range of characteristics, which include dental caries, dental plaque, dental calculus, gingival inflammation, missing teeth, and functional masticatory units. Plaque accumulation on tooth surfaces may affect mineralized and non-mineralized tissues within the mouth leading to the formation of dental caries and/or a chronic inflammation of the gingiva, which is the primary cause of periodontal diseases^1^,^2^. The worldwide prevalence of untreated caries in permanent teeth was 35% in 2010^3^, with almost half of adult Americans having periodontitis^4^.

Several observational studies have shown that periodontitis is associated with diseases in organs distal to the oral cavity, in particular cardiovascular disease^5^,^6^ and selected cancers^7^. Oral care has been shown to be inversely associated with mortality in individuals with tooth loss^8^. The inflammatory response associated with periodontitis^9^ has been suggested as a potential pathway to explain the link between oral and systemic health such that poor oral health is associated with high levels of low-grade inflammation^10^. It is also plausible that the bacteria of dental plaque can invade the surrounding connective tissue of the gingiva from which they reach the blood stream, creating a subsequent bacteremia^11^. These bacteria increase the blood levels of inflammatory mediators, such as lipopolysaccharides and cytokines^12^. It is also the case that periodontitis, as the main cause of tooth loss in middle aged and older adults^13^, impairs masticatory function when teeth are not replaced by dental prosthesis. This may lead to poor nutritional status, which has been associated with systemic disorders^14^. Having fewer than 10 teeth is significantly associated with malnutrition or obesity^15^,^16^.

While oral health as a whole is represented by an aggregation of an array of characteristics, a limited number of oral health variables have been used in previous reports of oral disease as a risk factor for health^17^. In addition, most studies were questionnaire-based^18^, rather than utilizing direct oral examination by a health professional. Accordingly, our study, based on clinical measurements, aims to evaluate the association between a combination of various oral health variables and future mortality using a propensity score model. Having previously found that poor oral health was associated with an elevated likelihood of hypertension^19^, we anticipated a similar relation with mortality.

## Methods

### Study population

The study population was retrieved from the “Centres d’Investigation Clinique et Preventive” (IPC) French cohort of volunteers, comprising 76,188 men and women who were aged between 16 and 89 years at recruitment. Described in detail elsewhere^19^,^20^ study members underwent a medical and dental examinations between January 2001 and December 2008 in medical centers (Centre d’Investigation Préventive et Clinique: IPC). Created in 1971 IPC are centers for health examination subsidized by the French National Health Insurance (*Caisse Nationale d’Assurance Maladie: CNAM*). They provide free medical and dental examinations every 5 years to workers, retirees, and their families living in Paris and the surrounding areas. We confirm that the data collection and the analysis were carried out in accordance with french guidelines and regulations. With the authorization from the Commission Nationale de l’Informatique et des Libertés (CNIL), data were anonymized prior to analyses. All volunteers provided informed consent for the use of all their recorded variables.

### Dental and periodontal examination

The participants received a full-mouth clinical examination from one of five trained dental examiners. A simplified plaque index based on that developed by Silness and Loe^21^ was used and included the following ratings: low (plaque cannot be seen with the naked eye), moderate (limited quantity of plaque can be seen), and high amount (abundance of soft matter within tooth and/or gingival margin). Similarly, the calculus index was rated: low (supragingival calculus covering no more than one-third of the tooth surface), moderate (supragingival calculus covering more than one-third but not more than two-thirds of the tooth surface and/or the presence of individual flecks of subgingival calculus around the cervical portion of the tooth), and high amount (supragingival calculus covering more than two-thirds of the tooth surface). Gingival inflammation was evaluated using a simplified Modified Gingival Index (MGI) based on that described by Lobene *et al*. The degree of gingival inflammation was rated as low (absence of inflammation or mild inflammation), moderate (inflammation, including the preceding criteria, in all portions of the gingival marginal or papillary tissue), or high (erythema, edema or spontaneous bleeding). A concordance rate between examiners was calculated and was 100% for dental calculus and masticatory efficiency, 86.7% for dental plaque, and 80% for gingival inflammation. A count of the number of teeth, with the exception of the third molars, was recorded. Masticatory ability was evaluated by the number of functional occlusal units defined by pairs of natural or prosthetic opposing premolars and molars.

### Covariate data

Body mass Index (BMI) was calculated as [weight (kg)/height squared (m2)]. Glycemia, total plasma cholesterol, and triglycerides were measured under fasting conditions. Diabetes was defined as a fasting glucose concentration of ≥126.0 mg/dl (7.0 mmol/l), a non-fasting glucose concentration of ≥200.0 mg/dl (11.1 mmol/l), or a self-reported history of treatmentfor diabetes. After ten minutes of rest, blood pressure measurements were made. Hypertension was defined as a systolic blood pressure ≥140 mm of mercury (mmHg) or diastolic blood pressure ≥90 mmHg. Alcohol consumption (daily consumption), cigarette smoking (former smokers, current smokers or non-smokers), and education level (two years college minimum or equivalent and less than two years) were self-reported using a detailed questionnaire.

### Mortality ascertainment

For each screened subject, vital status was obtained from the “Institut National de Statistiques et d’Etudes Economiques” (INSEE, Paris, France). Causes of mortality, taken from death certificates, were provided by INSERM’s Department of Mortality Studies (“Institut National de la Santé et de la Recherche Médicale”, Unit SC8). Causes of death were codified according to the International Classification of Disease (8th revision until 1978 and 9th revision until 2000 and 10 thereafter). To validate this procedure, in 2012 we compared a random sample of 364 subjects considered as deceased from our database and compared the mortality data with those provided by city hall registries for every candidate. Discordance was found in only two cases (0.55%). Furthermore, subjects with a personal medical history of cancer or cardiovascular disease and death by casualty were excluded from the analysis, representing 3079 subjects.

### Statistical analysis

The oral variables were dental plaque, dental calculus, gingival inflammation, tooth loss and occlusal units. For masticatory efficiency analysis, the participants were pooled into two groups: sufficient masticatory function versus insufficient masticatory function depending on the number of occlusal units (≥ or <5). Each oral variable was separately treated, and compared according to a dichotomic degree of exposure; that is, low versus high. Incidence rate was calculated by the ratio number of case/person-years. The statistical analysis was made in two stages. First stage, because of the large number of confounders regarding the sample size, and the inherent risk of overadjustment, propensity scores were calculated. The propensity score is a score based on the influence of covariates on the association between dental exposure and mortality. To generate propensity scores, multivariate logistic models were run in which each single oral health variable was the dependent variables (high dental plaque, high dental calculus, high gingival inflammation, number of teeth ≤10, number of functional occlusal units <5) and all others confounders (age, gender, body mass index, current smokers, diabetes, hypertension, gamma-glutamyl transferase (gamma_GT), cholesterol, and education level) were the independent variables. Scores were then calculated for each oral variables for each participant. The C-statistics for the propensity models are reported in Supplemental Data Table A. For each model, the C-statistic p-values were higher than 0.7 suggesting a high predictive value of the model. The second stage was to evaluate mortality risk using Cox regression model including the propensity score for each dental exposure. Mortality risks were evaluated for all-cause, cardiovascular, all-cancer, and non-cancer and non-cardiovascular mortality. Hazard Ratios for all-cause, all-cancer, and other causes of mortality were calculated for 0 to ≥3 oral health exposure. Statistical analyses were performed using the SAS statistical software (8.2 version; SAS Institute Inc., Cary, NC, USA).

## Results

During a follow-up time of 3.4 years (SD 2.4 years), a total of 370 person died; 184 deaths were due to cancer (0.24%) and 129 from non cardiovascular (non CV) or non cancer mortality (0.17%) among a sample size of 76188 patients. Table 1 shows the characteristics of the sample at baseline. The mean age was 44.9 ± 13.6 years. The table indicates that 0.5% of the subjects died. Compared to the subjects that were alive, the dead people had more cholesterol, and the number of diabetics was higher in this group than in the alive group. In addition, the people who died during the observation period have a higher consumption of alcool and cigarettes, a higher blood pressure, and a lower educational level compared those who were alive. No statistical difference between the two groups was found for BMI (p = 0.05).

**Figure 1:**
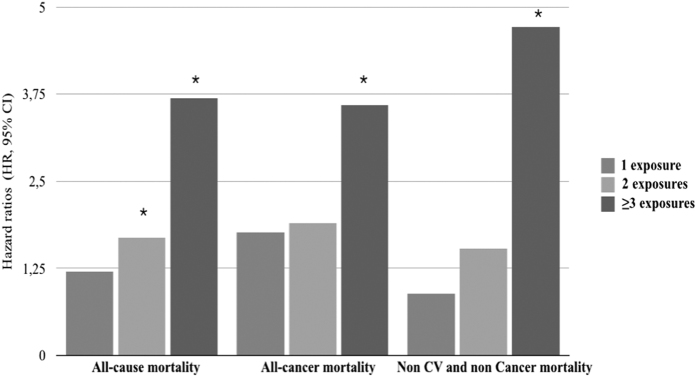
Histogram representing Hazard Ratios (HR, 95% CI) for all-cause, all-cancer, and non CV and non cancer mortality depending on cumulative dental exposure (dental plaque, dental calculus, gingival inflammation, functional Masticatory Units <5 and missing teeth >10) (Propensity score model).

Table 2 shows the incidence rate for all-cause, all-cancer and other causes of mortality according to oral health. When a high state was present (high dental plaque, high dental calculus, high gingival inflammation), the incidence rates were higher for every causes of mortality compared to the low/moderate status. Similar results were found when more than 10 teeth were missing or when functional masticatory units were below 5. When 3 or more dental exposures were present, the incidence rates of all-cause mortality, all-cancer mortality, and non cardiovascular and non cancer mortality were, 2.26 (CI 95%: −0.82; 5.34), 3.67 (CI 95%: −0.25; 7.59), 6.22 (CI 95%: 1.12; 11.31) respectively (Supplemental Data Table B). In a propensity score adjustment model, high state of all dental variables, except dental calculus, were positively associated with all-cause, non cardiovascular and non cancer mortality. All-cancer mortality was positively associated with dental plaque and gingival inflammation only (Table 3). The risk of mortality, in case of cumulative oral disease was also explored. Compared to the reference group, an increased significant hazard ratio for every cause of mortality was found when 3 or more dental exposure were included in the model. The significance for all-cause of mortality risk exists when only 2 dental exposures were cumulated (Table 4) (Fig. 1).

## Discussion

### Main findings

In the present study, using a propensity score that has not, to the best of our knowledge, been previously utilized, we show a significant, positive association between poor oral status and mortality. It demonstrates, for the first time, that the accumulation of oral disease is associated with all-cause mortality, all-cancer mortality and non cancer and non cardiovascular mortality. We also found a positive linear association of dental exposures on all-cause mortality, such that there was a stepwise relation between poor oral health and higher risk of death.

#### Oral status and all-cause mortality

Our results indicate that missing teeth and insufficient masticatory function are associated with all-cause mortality. This association could be explained by eating habits. The link between food choices and masticatory efficiency is well established^14^. It has been shown that dietary fiber intake was lower among subjects with reduced masticatory function^23^. Therefore, it could be that tooth loss impairs masticatory function and this leads to poor diet. On the other hand, it has been demonstrated that lifestyle habits in particular dietary are strongly related to lifetime mortality^24^. This may explain the relationship between poor masticatory function and mortality.

In the IPC cohort study 0.5% of study members had dies (n = 370). This is in accordance with the most recent data on the premature causes of death in France. In addition, subjects included in the IPC cohort are volunteers, and have a healthcare insurance that cover free follow-up medical examination every 5 years. One can assume that these subjects are well concerned by healthcare.

#### Oral status and all-cancer mortality

The present findings show that dental plaque and gingival inflammation are associated with all-cause cancers mortality whereas other oral disease variables were not linked to cancer. A relationship between dental plaque and cancer has been previously demonstrated^7^. Furthermore, it has been suggested that oral bacteria and inflammation may play a role in carcinogenesis^25^ even if further studies are needed to highlight the pathophysiological pathway. In contrast, no significant correlation has been reported between missing teeth and cancer mortality^26^,^27^,^28^.

#### Study strengths and limitations

These study have several strengths. To our knowledge, it is the first report showing an association between oral health and mortality using cumulative exposure to dental variables. The study population is a large in size and members took part in a comprehensive medical examination. The results are close to those of the “CepiDC 2008” (National epidemiologic register of causes of death in France). Finally, the use of propensity-score analysis is well-adapted to an epidemiological study including a large number of confounding variables. This approach, which is not common in oral epidemiology, reduces the risk of overadjustment.

A limitation of the present study is the inability to analyze the association with cardiovascular mortality because of the middle-age of the cohort where cardiovascular fatal events were rare (0.08% of the sample). In this study all-cause of cancer mortality were included. In addition, the small sample size of cancer death (n = 184) did not allowed robust conclusions according to site-specific cancers. Taken together the results of previous studies dealing with the association between site-specific cancers and missing teeth are conflicting^28^,^29^. It has been shown that lung cancer is not associated with tooth loss^27^,^28^,^29^,^30^, whereas pancreas cancer mortality^31^, and orodigestive cancers^32^ have been positively associated with it. Further well designed studies aiming to identify the relationship between tooth loss and particular types of cancer are needed. It cannot be discarded that the wide age range of the study participants (16–89 years old) causes inflation of hazard ratios. We thus conducted an analysis stratified by several age group (Supplemental Data Table C). No variation between the different age classes was found, except for dental calculus; which becomes significant in the 50–59 age group. It seems that in subjects of 60 years and over, even if the associations are still significant the relationship seems to decrease. Finally, because the present study is observational, residual confounding is likely. Even if the external validity of a convenient sample can be challenged, large sample size, such as those of the IPC cohort (n = 76.188), can be useful to introduce new hypotheses and support future clinical trials. A clinical trial showing the impact of the treatment of poor oral health on mortality risk would be optimal. The feasibility of this type of study is unlikely. However, studies have been already conducted in the context of systemic inflammation. For example, previous reports have shown that the reduction of oral inflammation may have an impact on fatal diseases such as cardiovascular disease^9^.

In conclusion, we demonstrate that the accumulation of dental exposures is associated with mortality. Our results highlight the impact of oral health on general health, and support preventive measures at the population level.

## Additional Information

**How to cite this article**: Adolph, M. *et al*. Oral health in relation to all-cause mortality: the IPC cohort study. *Sci. Rep.* **7**, 44604; doi: 10.1038/srep44604 (2017).

**Publisher's note:** Springer Nature remains neutral with regard to jurisdictional claims in published maps and institutional affiliations.

## Supplementary Material

Supplemental Datasets

## Figures and Tables

**Table 1:** Baseline characteristics according to vital status.

—	Alive	All-cause mortality	p-values^a^	All-cancer mortality	p-values^a^	Non CV and non cancer mortality	p-values^a^	Total cohort
Population, n (%)	75818 (99.5)	370 (0.5)		184 (0.24)		129 (0.17)		76188 (100.0)
Male, n(%)	48598 (64.0)	298 (80.5)		146 (79.3)		106 (82.1)		48896 (64.0)
Age, y^b^	44.7 ± 13.5	59.4 ± 12.7	<0.001	59.9 ± 10.8	<0.001	57.0 ± 14.0	<0.001	44.9 ± 13.6
BMI (kg/m^2^)^b^	25.2 ± 4.4	26.0 ± 4.9	0.05	25.5 ± 4.1	0.03	26.4 ± 6.0	0.77	25.2 ± 4.2
Cholesterol (mg/dl)^b^	207.7 ± 41.4	218.9 ± 42.8	0.002	219.4 ± 41.8	0.02	216.5 ± 44.8	0.08	207.8 ± 38.4
Diabetes, n (%)	2721 (3.6)	44 (11.9)	<0.001	24 (13.0)	0.001	13 (10.1)	<0.001	2765 (3.6)
SBP (mm Hg)^b^,^c^	129.4 ± 18.7	146.8 ± 25.1	<0.001	144.6 ± 22.0	<0.001	145.9 ± 25.2	<0.001	129.4 ± 16.9
DBP (mm Hg) b,c	77.2 ± 11.8	85.2 ± 14.1	<0.001	84.3 ± 12.1	0.026	84.7 ± 14.8	0.004	77.3 ± 10.9
Hypertension, n (%)	14246 (18.8)	166 (44.9)	<0.001	80 (43.5)	<0.001	57 (44.2)	<0.001	14412 (18.9)
Gamma_GT	34.8 ± 51.4	85.2 ± 218	<0.001	69.6 ± 32.8	<0.001	89.6 ± 164.1	<0.001	35.02 ± 52.1
Smokers, n (%)	21466 (28.3)	155 (41.9)	<0.001	78 (42.4)	<0.001	60 (46.5)	<0.001	21621 (28.4)
Education.level, n (%)^d^	29412 (38.8)	103 (27.8)	<0.001	53 (28.8)	0.01	34 (26.4)	0.01	29515 (38.7)
Alcohol consumption (glass/day)^b^	0.71 ± 1.6	2.08 ± 2.90	<0.001	2.13 ± 2.51	<0.001	2.19 ± 3.33	<0.001	0.72 ± 1.6

^a^All p values were calculated versus alive.

^b^Data are show as mean ± SD.

^c^Hypertension was defined as systolic blood pressure (SBP) higher than 140 mmHg and/or diastolic blood pressure (DBP) higher than 90 mmHg.

^d^Two years college or equivalent and more.

**Table 2:** Incidence rate for all-cause, all-cancer and non CV and non cancer mortality according to oral health.

	N	Person-year	All-cause mortality (n = 370)			All-cancer mortality (n = 184)			Non CV and non cancer mortality (n = 129)		
			Number of cases	Incidence rate (‰)	95% CI	Number of cases	Incidence rate (‰)	95% CI	Number of cases	Incidence rate (‰)	95% CI
**Dental Plaque**											
Low	66 590	229 070	285	1.24	[0.97;1.51]	145	0.63	0.43;0.83	94	0.41	[0.25;0.57]
Moderate	8804	30 286	57	1.88	[0.92;2.84]	25	0.83	[0.21;0.51]	61	0.69	[−0.31;1.69]
High	1627	5955	28	4.70	[1.29;8.11]	14	2.35	[−0.06;4.76]	14	2.35	[−0.06;4.76]
**Dental Calculus**											
Low	23 582	75 462	106	1.40	[0.87;1.92]	47	0.62	[0.27;0.97]	41	0.54	[0.21;0.87]
Moderate	46 873	170 618	200	1.17	[0.86;1.48]	109	0.64	[0.41;0.88]	60	0.35	[0.17;0.53]
High	6566	19 173	64	3.34	[1.73;4.95]	28	1.46	[0.4;2.52]	28	1.46	[0.4;2.51]
**Gingival inflammation**											
Low	52 235	170 808	192	1.12	[0.81;1.43]	97	0.57	[0.35;0.79]	66	0.39	[0.21;0.57]
Moderate	21 299	81 788	123	1.50	[0.97;2.03]	64	0.78	[0.41;1.15]	38	0.47	[0.18;0.76]
High	3487	12 274	55	4.48	[2.13;6.83]	23	1.87	[0.36;3.38]	25	2.04	[0.47;3.61]
**Missing Teeth**											
≤10	60 165	229 070	221	1.07	[0.82;1.32]	114	0.55	[0.37;0.73]	76	0.37	[0.23;0.51]
>10	5836	16 808	93	5.53	[3.33;7.73]	39	2.39	[0.96;3.82]	35	2.08	[0.73;3.43]
**Functional Masticatory Units**											
≥5	70 313	246 096	301	1.22	[0.95;1.49]	157	0.64	[0.44;0.84]	96	0.39	[0.23;0.55]
<5	6708	19 400	69	3.57	[1.92;5.22]	27	1.41	[0.39;2.43]	33	1.73	[0.39;3.06]

**Table 3:** Hazard Ratios (HR, 95%) for all-cause, all-cancer and non CV and non cancer mortality in case of High Amount of Dental Plaque, Dental Calculus, Gingival Inflammation and Masticatory Efficiency status (Propensity score model).

	All-cause mortality	All-cancer mortality	Non CV and non cancer mortality
Dental Plaque	2.73 (2.19–3.40)	2.36 (1.32–4.22)	3.30 (1.76–6.17)
Dental Calculus	1.12 (0.92–1.38)	1.16 (0.70–1.93)	1.03 (0.59–1.77)
Gingival Inflammation	1.68 (1.38–2.05)	1.92 (1.18–3.12)	2.86 (1.71–4.79)
Missing teeth >10	2.02 (1.73–2.37)	1.49 (0.95–2.34)	2.31 (1.40–3.82)
Functional Masticatory Units <5	1.96 (1.68–2.29)	1.15 (0.74–1.79)	2.40 (1.55–3.73)

**Table 4:** Hazard Ratios (HR, 95% CI) for all-cause, all-cancer, and non CV and non cancer mortality depending on cumulative dental exposure (dental plaque, dental calculus, gingival inflammation, functional Masticatory Units <5 and missing teeth >10) (Propensity score model).

Dental exposure	All-cause mortality n = 370	All-cancer mortality n = 184	Non CV and non cancer mortality n = 129
0 n = 15222 (reference group)	1	1	1
1 n = 4242	1.21 (0.88–1.67)	1.77 (0.76–4.09)	0.89 (0.36–2.20)
2 n = 1600	1.69 (1.16–2.46)	1.90 (0.72–5.0)	1.54 (0.54–4.4)
≥3 n = 1022	3.69 (2.51–5.42)	3.59 (1.23–10.5)	4.71 (1.74–12.7)
Increase of 1 dental exposure	1.71 (1.62–1.81)^b^	1.56 (1.34–1.81)^b^	1.86 (1.49–2.16)^b^
