# Supplementary material for: Oral health in relation to all-cause mortality: the IPC cohort study
Source: Sci Rep. 2017 Mar 15;7:44604. doi: 10.1038/srep44604 (PMC5353629; doi:10.1038/srep44604)
Supplement: Supplemental Datasets [file srep44604-s1.pdf]

Oral health in relation to all-cause mortality: the IPC cohort study

Margaux Adolph, DDS [1 +], Christelle Darnaud, DDS [1 +], Frédérique Thomas, PhD[2], Bruno Pannier, MD, PhD[2,3], Nicolas Danchin, MD, PhD[2,4], G. David Batty, DSc, PhD[5], and Philippe Bouchard, DDS, PhD* [1,6]

[1]Department of Periodontology, Service of Odontology, Rothschild Hospital, AP-HP, Paris 7-Denis Diderot University, U.F.R. of Odontology,Paris, France;
[2]Centre d'Investigation Préventive et Clinique (IPC), Paris, France;
[3]Manhès Hospital, Fleury-Mérogis, France;
[4]Department of Cardiology, Georges Pompidou European Hospital, AP-HP, Paris 5 - Descartes University, Medicine Faculty, Paris, France;
[5]Department of Epidemiology and Public Health, University College London, London, UK
[6]EA 2496, Paris 5 - Descartes University, U.F.R. of Odontology, Paris, France.

[+] Margaux Adolph and Christelle Darnaud contributed equally to this work

Supplemental Materials

Table A: Average values of propensity scores for each oral variable

	**Mean**	**Minimum**	**Maximum**	**C statistic**
**Dental Plaque**	0.024	0.001	0.492	0.786
**Dental Calculus**	0.227	0.02	0.91	0.731
**Gingival Inflammation**	0.065	0.006	0.67	0.737
**Missing teeth >10**	0.085	0.001	0.91	0.855
**Functional Masticatory Units <5**	0.083	0.008	0.63	0.756

Table B: Incidence rate for all-cause, all-cancer and non CV and non cancer mortality according to dental exposure

			All-cause mortality (n=370)			All-cancer mortality (n=184)			Non CV and non cancer mortality (n=129)		
**Dental exposure**	N	Person-year	Number of cases	Incidence rate (‰)	95% CI	Number of cases	Incidence rate (‰)	95% CI	Number of cases	Incidence rate (‰)	95% CI
0	15222	49167	17	0.35	[0.04;0.66]	16	0.33	[0.02;0.64]	36	0.73	[0.26;1.2]
1	4242	9969	16	1.61	[0.06;3.16]	13	1.30	[-0.09;2.69]	37	3.71	[1.36;6.06]
2	1600	5264	11	2.09	[-0.32;4.5]	8	1.52	[-0.54;3.6]	24	4.56	[0.99;8.13]
>3	1022	3536	8	2.26	[-0.82;5.34]	13	3.67	[-0.25;7.59]	22	6.22	[1.12;11.31]

Table C: Hazard Ratios (HR, 95%) for all-cause mortality in case of High Amount of Dental Plaque, Dental Calculus, Gingival Inflammation and Masticatory Efficiency status, according to age group (Propensity score model)

	All-cause mortality	<30	30-49	50-59	>=60
**Dental Plaque**	2.73<br>(2.19-3.40)<br>P<0.0001	NA	3.23<br>(2.06-5.08)<br><0.0001	3.51<br>(2.51-4.91)<br><0.0001	1.94<br>(1.26-2.99)<br>0.003
**Dental Calculus**	1.12<br>(0.92-1.38)<br>P=0.26	NA	1.15<br>(0.73-1.81)<br>ns	1.50<br>(1.08-2.09)<br>0.017	1.24<br>(0.89-1.72)<br>Ns
**Gingival Inflammation**	1.68<br>(1.38-2.05)<br>P<0.0001	NA	2.84<br>(1.94-4.18)<br><0.0001	1.90<br>(1.73-2.59)<br><0.0001	1.49<br>(1.08-2.05)<br>0.0005
**Functional Masticatory Units <5**	1.96<br>(1.68-2.29)<br>P<0.0001	NA	2.68<br>(1.86-3.88)<br><0.0001	2.005<br>(1.54-2.62)<br><0.0001	1.46<br>(1.18-1.81)<br>0.0005
**Missing teeth >10**	2.02<br>(1.73-2.37)<br>P<0.0001	NA	1.75<br>(1.24-2.43)<br>0.0012	2.11<br>(1.60-2.72)<br><0.0001	1.95<br>(1.52-2.51)<br><0.0001
